# Supplementary material for: Mechano-gated iontronic piezomemristor for temporal-tactile neuromorphic plasticity
Source: Nat Commun. 2025 Jan 26;16:1060. doi: 10.1038/s41467-025-56393-w (PMC11770186; doi:10.1038/s41467-025-56393-w)
Supplement: Supplementary file 1 — Supplementary Information [file 41467_2025_56393_MOESM1_ESM.pdf]

Supplementary Information

Mechano-gated Iontronic Piezomemristor for Temporal-tactile Neuromorphic Plasticity

Xiao Wei[1,2,3†], Zhixin Wu[1,2†], Hanfei Gao[3†], Shiqi Cao[4†], Xue Meng[1,2], Yuqun Lan[5], Huixue Su[1,2], Zhenglian Qin[1,2], Hang Liu[1,2], Wenxin Du[6], Yuchen Wu[1,2,3*], Mingjie Liu[6*], Ziguang Zhao[1,2*]

[1]School of Future Technology, University of Chinese Academy of Sciences, Beijing 100190, P. R. China.
[2]Key Laboratory of Bio-inspired Materials and Interfacial Science, Technical Institute of Physics and Chemistry, Chinese Academy of Sciences, Beijing 100190, P. R. China.
[3]Suzhou Institute for Advanced Research, University of Science and Technology of China, Suzhou 215123, Jiangsu, P. R. China.
[4]Orthopaedics of TCM Senior Department, the Sixth Medical Center of Chinese PLA General Hospital, Beijing 100048, P.R. China.
[5]State Key Laboratory of Nonlinear Mechanics, Institute of Mechanics, Chinese Academy of Sciences, Beijing 100190, P. R. China.
[6]School of Mechanical Engineering and Automation, Beihang University, Beijing 100191, P. R. China.

†These authors contributed equally to this work.
*Corresponding author. Emails: wuyuchen@iccas.ac.cn; liumj@buaa.edu.cn; zhaoziguang@ucas.ac.cn.

The file includes:

Supplementary Text:

Materials

Preparation of the vitrimers

Preparation of the ion-liquid gels

Preparation of the mixed solution of PEDOT:PSS

Fabrication of the iontronic memristor

Fabrication of reconfigurable mechanogate

Nano CT characterization

AFM characterization

Mechanical measurements

Rheological test

Piezoresistivity measurement

Infrared thermography measurement

Device characterization

The learning rules in the MIPM system

Device operating mechanism

Finite element modeling

Supplementary Figures 1 to 27

Supplementary Table 1

Supplementary References

Supplementary Text:

Materials

Poly(caprolactone) diol (PCL-diol, Mn ~ 1000), tri-functional homopolymer of hexamethylene diisocyanate (THDI, Desmodur® N 3900), and hexafluorobutyl acrylate (HFBA) was purchased from Sigma-aldrich. Ethyleneglycol dimethacrylate (EGDMA) and phenylbis (2,4,6-trimethylbenzoyl) phosphine oxide (PBPO, photoinitiator) were purchased from J&K Chemical Ltd., China. Dibutyltin dilaurate (DBTDL) and toluene were purchased from Aladdin. Ionic liquid (IL) was purchased from Lanzhou Yulu Fine Chemical Co., Ltd. (GanSu, China). PEDOT:PSS (Clevios PH1000) were purchased from J&K Chemical Ltd., China. 4-dodecylbenzenesulfonic acid (DBSA) and (3-glycidyloxypropyl) trimethoxysilane were purchased from Sigma-aldrich. All chemicals were used without any further purification unless otherwise noted.

Preparation of the vitrimers

The vitrimers were synthesized through a polyurethane reaction. 2 g of PCL-diol, 0.3 g of toluene, DBTDL and 0.06 g of THDI were combined and stirred for several minutes. The resulting mixture was poured into a mold and subjected to thermal curing at 80°C for 24 hours. Finally, the cured sample was placed at 80°C for 24 hours.

Preparation of the ion-liquid gels

1 g of IL, 1 g of HFBA, and 0.001 g PBPO were mixed and stirred to generate a stable precursor solution. Next, ILgels were prepared using the UV (405 nm) free-radical polymerization for 4 hours at room temperature.

Preparation of the mixed solution of PEDOT:PSS

The PEDOT:PSS aqueous dispersion was mixed with 5 vol% ethylene glycol to enhance their conductivity, 0.25 vol% DBSA to improve their homogeneity, and 1 vol% (3-glycidyloxypropyl) trimethoxysilane as a crosslinking agent. The solution was stirred continuously for 48 hours at room temperature to ensure thorough mixing of the components.

Fabrication of the iontronic memristor

To fabricate high-quality PEDOT:PSS channel, an interface-confined assembly strategy based on capillary bridges employing periodic line-shaped micropillar templates with asymmetric wettability was used to manipulate the dewetting dynamics. In this assembling system, a continuous layer of liquid was pinned initially between the substrate and the lyophilic micropillar tops. With solvent evaporation, the continuous liquid layer ruptured into the isolated capillary bridges anchored onto the tops of the pillars, which is driven by the difference of Laplace pressure. Further control the spreading of the three-phase current line, a confined space between the tops of micropillars and the target substrate is generated. Once the PEDOT:PSS

solution reached saturation concentration, molecules begin to self-assemble in the confined interface. The confined space with microscaled width and nanoscaled height will terminate the horizontal and vertical assembly while continue to grow along with the length of micropillars, facilitating one-dimensional (1D) self-assembly of molecules. After the exhaustion of solvent, the PEDOT:PSS microwire arrays with precisely controlled geometry and position were fabricated on the target substrate (between source electrode and drain electrode). For the iontronic memristor within the MIPM, PEDOT:PSS channels connect with the oscillating digital circuit through an ionic conductive gel.

Fabrication of reconfigurable mechanogate

The reconfigurable mechanogate with different surface patterns were fabricated using patterned Polytetrafluoroethylene (PTFE) molds via hot pressing. The heterogel samples were hot pressed between PTFE molds at 140°C for 6 h to activate the dynamic covalent networks in the vitrimer phase. The original smooth surface could be restored by hot pressing the patterned samples with smooth PTFE molds under the same conditions (140°C, 6 h).

Nano CT characterization

A high-resolution Nano CT device (Bruker SkyScan2214CMOS, Germany) was used to scan the specimens at a voxel size of 100 nm, 60 kV, 100 µA, 360° of rotation, a

0.08° of rotation step, 4 of averaging frames. The bicontinuous heterogels were cut to the specific size (800 μm height × 800 μm width × 1 mm thickness).

AFM characterization

The MultiMode 8 atomic force microscope (AFM) with NanoScope V controller from Bruker (Bruker Corporation, Santa Barbara, CA) equipped with PeakForce Quantitative Nanomechanical Mapping (PF-QNM) mode was used for samples topography imaging and nanomechanical mapping under ambient conditions. NPS (Bruker′s antimony (n) doped silicon probes with a nominal spring constant of 40 N m^{-1} and a tip radius of 10 nm) type probes were chosen. In PF-QNM mode AFM, the deflection sensitivity and the spring constant for each cantilever were calibrated using the built-in cantilever calibration, the ramp, and thermal noise method, respectively. All AFM experiments were performed under ambient conditions.

Mechanical measurements

The compressive tests was conducted using a tensile-compressive tester (Mark-10/ESM301). Cylinder-shaped samples (5 mm diameter × 10 mm height) were used for compressive tests with a deformation rate of 10% of the sample height per minute.

Rheological test

The rheological properties of the samples were investigated by a modular compact rheometer (Anton Paar, MCR 301). For frequency sweeps, a 15 mm parallel plate geometry was used with a 1 mm gap size at a constant temperature of 40°C and strain of 0.1%. Under a variety of temperatures, the storage modulus (G') of the samples was swept in a range of 0.1 ~ 100 rad s^{-1} or at 15.8 rad s^{-1} at a constant strain of 0.1 %.

Piezoresistivity measurement

The piezoresistivity performance of the bicontinuous heterogels was evaluated on a homemade test system consisting of a forcemeter (Mark-10/ESM301) and electrochemical workstation (CH Instruments, CHI760E). We tested the piezoresistivity performance of bicontinuous heterogels with both the 25 and 40°C through the amperometric i-t test model.

Infrared thermography measurement

The infrared thermography performance of the bicontinuous heterogels was evaluated using a customized test setup comprising an infrared thermal imaging camera (FLIR T650sc) and a programmable heating platform. The surface temperature distribution of the heterogels was recorded in real-time under controlled heating at 40°C, 50°C and 60°C.

Device characterization

The MIPM system was powered by a 6.5 V DC source (either an electrochemical workstation or a button cell battery). Standard weights were used to apply pressure. The pressure signals were converted to current signals, which were subsequently pre-amplified. Based on the processed current signals, an oscillation circuit generated positive or negative oscillating voltages of varying frequencies, serving as the gate voltage for the iontronic memristor (positive voltage: 0.4 V, negative voltage: -0.8 V). The source-drain voltage was maintained at a constant -0.5 V. The post-synaptic current signals outputted by the device were measured using the i-t mode of a Keysight B1500A semiconductor analyzer.

The learning rules in the MIPM system

In the MIPM system, the BCM rule is realized through programmed temporal pressure stimulations on two heterogel mechanogates in different states in parallel. We conducted three controlled trials of gradient-increased pressure on the soft-state mechanogate as history activities, followed by three constant pressures on the stiff-state mechanogate as testing trials. The three sets of spike trains (successive positive spikes) at gradient-increased frequencies, corresponding to the three sets of history pressure, resulted in increasing inhibitory plasticity and adjusted the MIPM's the long-term memory to different levels below the resting current.

Device Operating Mechanism

In the MIPM system, the conversion of bidirectional piezoresistive signals into positive and negative pulse trains is accomplished by the microcontroller unit (MCU). The process involves several components: The pre-amplifier, connected to the mechanogate, receives and amplifies the bidirectional piezoresistive signal inputs. These amplified signals are then sent to the microcontroller, where they are continuously sampled through the ADC module and processed by the CPU. The conversion process relies on a differential threshold mechanism, where the system calculates the difference between consecutive ADC measurements to determine the signal direction and magnitude. When this difference exceeds a positive threshold, the system generates positive pulse trains, while differences below a negative threshold produce negative pulse trains. The microcontroller's RCC (Reset and Clock Control) generates oscillatory pulses based on the encoded signals. The amplitude of these spike pulses is fixed, with positive spike trains maintaining a constant amplitude of 0.4 V and negative pulse trains at -0.8 V. The frequency of the pulses is determined by a specific linear relationship with the ADC value, following the equation: PWM_PRD = -29.2864 * AD_FINAL_VAULE + 113945.428 or PWM_PRD = 48.81855 * AD_FINAL_VAULE + 5000. This relationship operates within carefully designed constraints, including a PWM frequency range of 8 Hz to 100 Hz and an analog input range of 0 V to 3 V. This comprehensive signal processing approach ensures that temporal and intensity information from the original piezoresistive signals is preserved through frequency modulation, while the amplitude is

standardized to fixed values. The following code demonstrates the implementation and execution of this process. Next, these positive and negative spike trains are further transmitted to downstream memristors to modulate the output of bilingual neuromorphic signals.

The code is provided as follows:

```
/*******************************
 *
 * New Demond
 *
 ******************************/
If (TIM16_OUT_TRIG == 1) //ADC Final Count & generate PWM
{
        AD_FINAL_VAULE = AD_VAULE_ADDED / AD_COUNT;
        AD_COUNT = 0;
        AD_VAULE_ADDED = 0;

        /* AD RANGE 0 to +3V */
        /* AD VALVE RANGE 0-3720 */
        /* AD VALVE COMPARE -120nA to 0V */
        /* AD VALVE COMPARE 0nA to 1V */
        /* AD VALVE COMPARE 240nA to 3V */
        /* PWM Frequency RANGE 7.63Hz-100Hz */
        /* PWM_PRD Range 5000-65535 */
        If (AD_FINAL_VAULE >= 1653)    //40nA
        {
              PMW_DIR = 1; //Pos State
              PWM_REGENERATE = 1;
        }
        else if (AD_FINAL_VAULE << 1240)
        {
              PMW_DIR = 0; //Neg State
              PWM_REGENERATE = 1;
        }
        TIM16_OUT_TRIG = 0;
}
If (PWM_REGENERATE == 1)
{
         HAL_TIM_Base_Stop_IT(&htim3);
         HAL_TIM_Base_DeInit(&htim3);
         HAL_GPIO_WritePin(GPIOB,GPIO_PIN_6,GPIO_PIN_RESET);
         PWM_Output_L_State = 0;
         HAL_GPIO_WritePin(GPIOB,GPIO_PIN_5,GPIO_PIN_RESET);
         PWM_Output_H_State = 0;
         If (PMW_DIR == 1)      PWM_PRD = -29.2864 * AD_FINAL_VAULE + 113945.428 ;
         If (PMW_DIR == 0)      PWM_PRD = 48.81855 * AD_FINAL_VAULE + 5000 ;
         MX_TIM3_Init();
         HAL_TIM_Base_Start_IT(&htim3);
         PWM_REGENERATE = 0;
}
AD_VAULE_LAST = AD_FINAL_VAULE;
}
```

Finite element modeling

Finite element modeling (COMSOL Multiphysics6.0, MA, USA) was employed to simulate the ionic transport pathway of the bicontinuous phase structure under deformation, the 3D model was built from 3DS MAX 2020 (Autodesk, USA). In the

simulation setup, Young's modulus of stiff vitrimer at room temperature and high temperature were set as 208 MPa and 74 kPa, and Poisson ratios were 0.1 and 0.3 with the same density which was 1.1 g/ml. Young's modulus of IL-gel was 1.8 kPa at both room temperature and high temperature, that Poisson ratio was 0.3, and the density was 1.46 g/ml. The overall shape variable of the structure is set to 50%. Our simulation results indicate that in the stiff state, the high modulus of vitrimer phase restricts lateral deformation of IL-gel phase. Consequently, axial compression primarily shortens the ion transmission pathways, slightly increasing the effective cross-sectional area of the IL-gel phase with ion channels from 49.4% to 49.9% under applied pressure. This increase in cross-sectional area, coupled with shortened ion channels, is consistent with our experimental observations of negative piezoresistivity. Conversely, in the soft state, the significantly reduced modulus of vitrimer allows for substantial lateral expansion of heterogel materials under compression. This lateral deformation causes the ILgel phase to become partially interrupted and disconnected, resulting in a substantial decrease in the effective cross-sectional area from 49.4% to 31.2%. These simulation results align well with our observations of positive piezoresistivity and support the proposed mechanisms.

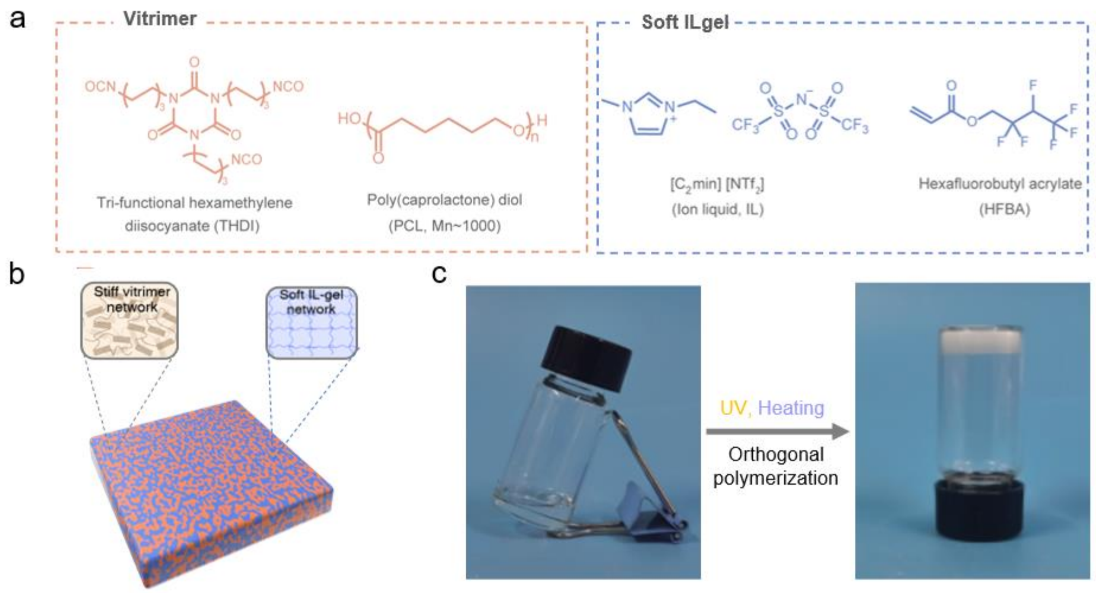

Supplementary Fig. 1 (a) Chemical structures of reacted precursor components within bicontinuous heterogels. **(b)** The heterogels have bicontinuous structure of vitrimer and ILgel framework phases. **(c)** Preparation of the heterogels through orthogonal polymerizations.

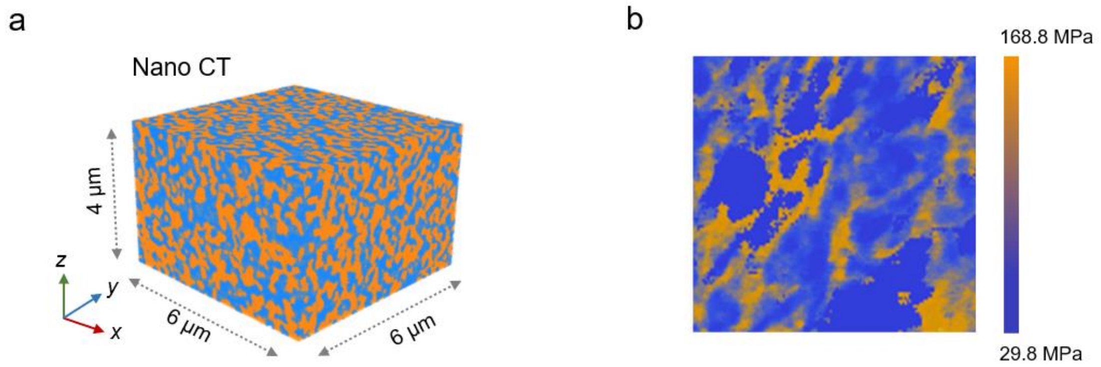

Supplementary Fig. 2 (a and b) Nano CT image and AFM measurement of the bicontinous heterogel.

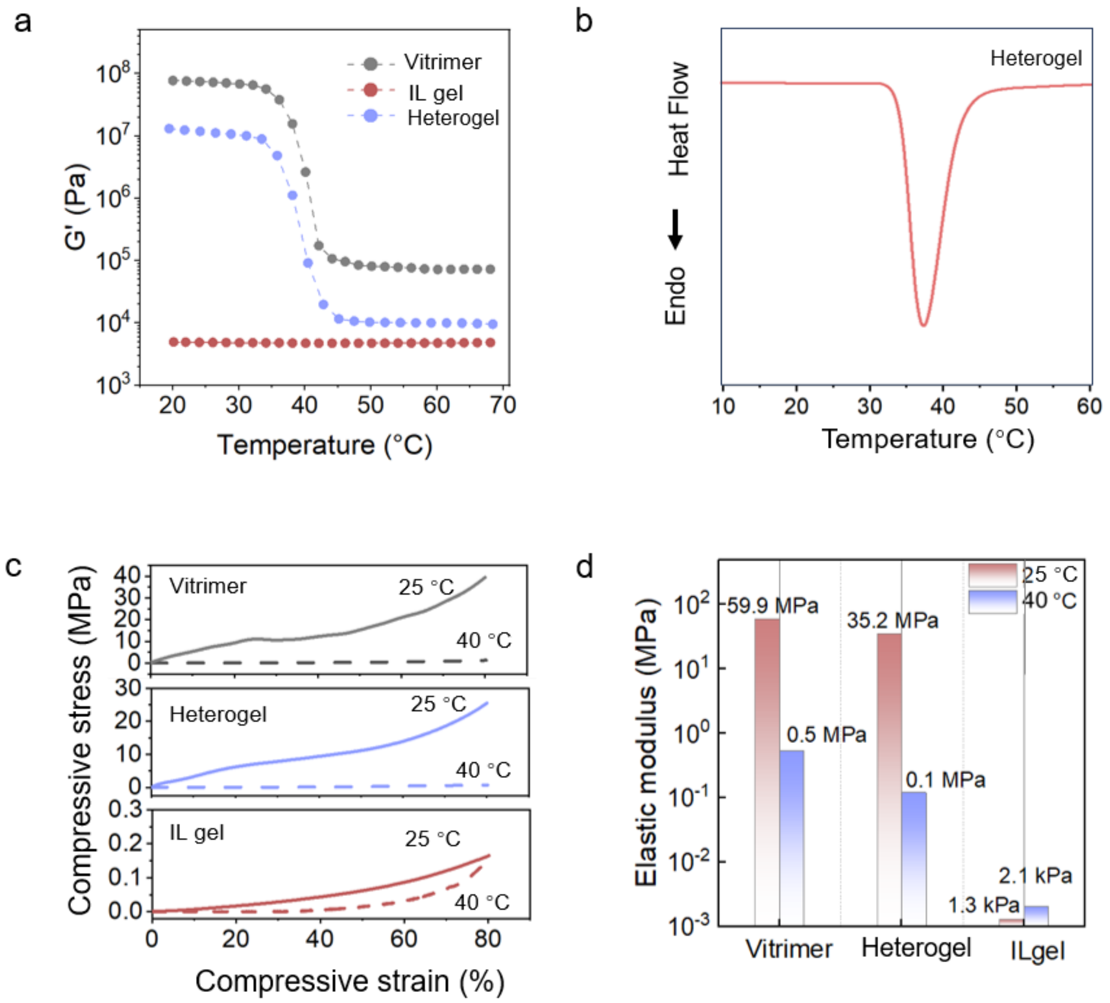

Supplementary Fig. 3 (a) Storage moduli (*G*') of the vitrimer, heterogels, and the ILgel on a temperature sweep in the range of 20 to 40°C**. (b)** Differential scanning calorimetry (DSC) thermogram of the heterogels on a temperature sweep in the range of 10 to 60°C. **(c and d)** Compressive stress-strain curves and elastic modulus of the heterogel, vitrimer and ILgels at 25°C and 40°C.

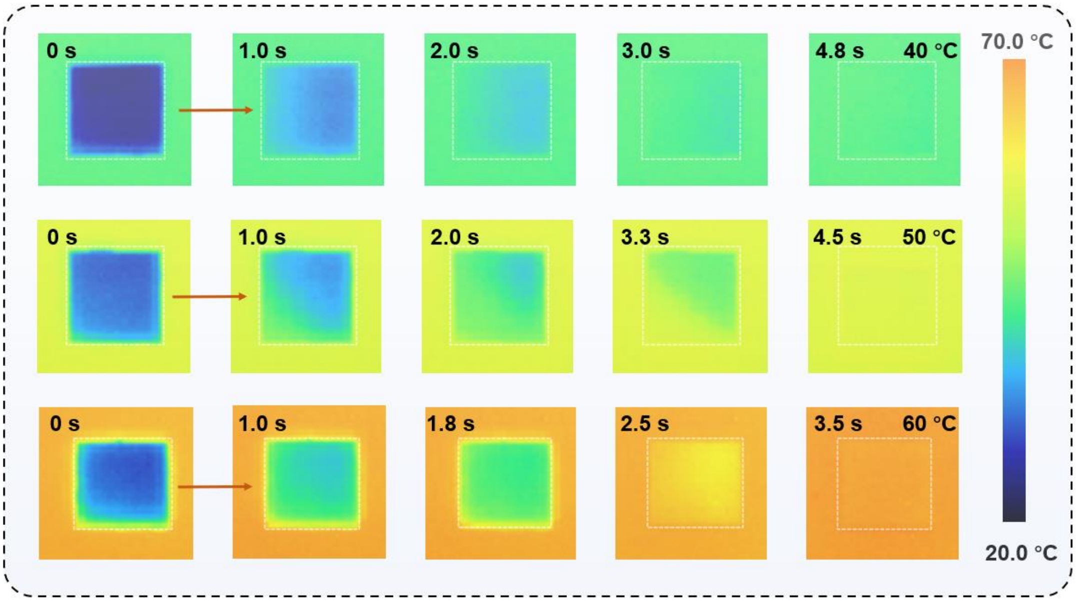

Supplementary Fig. 4 Infrared thermography showing the heat conduction process of heterogel films with the thickness of 200 μm at 40°C, 50°C, and 60°C. The color mapping represents temperature change.

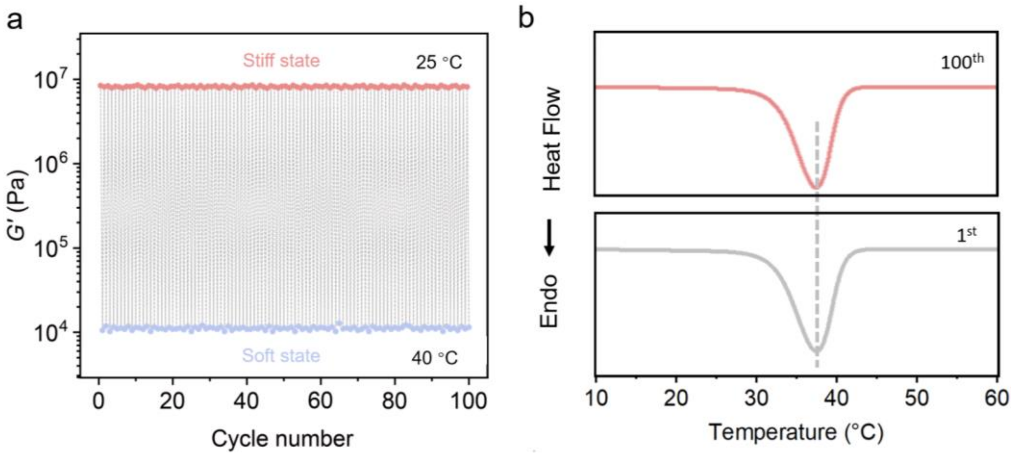

Supplementary Fig. 5 (a) Stable transitions between high and low *G*' of heterogels at 100 continuous switching cycles between stiff (25°C) and soft (40°C) states. **(b)** Differential scanning calorimetry (DSC) thermograms comparing the 1st and 100th thermal cycles across the temperature range from 10 to 60 °C.

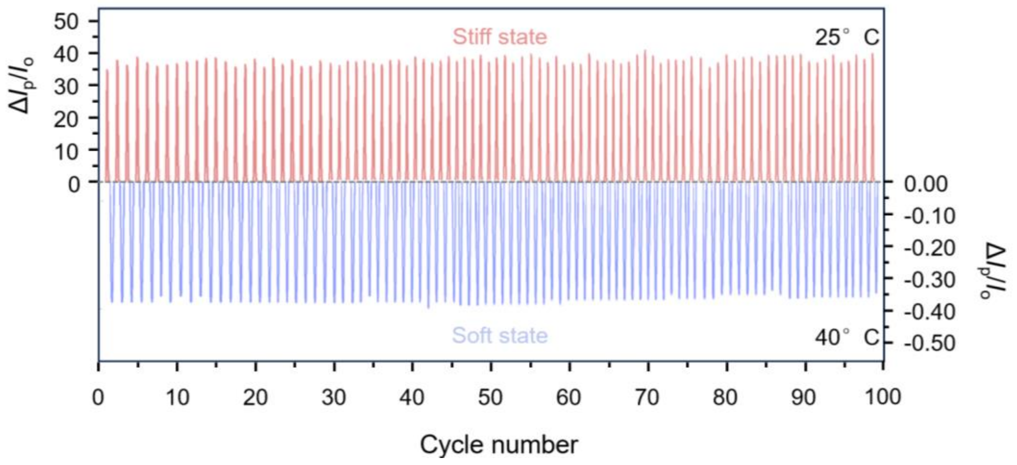

Supplementary Fig. 6 The stable bidirectional piezoresistive performance of the bicontinuous heterogels.

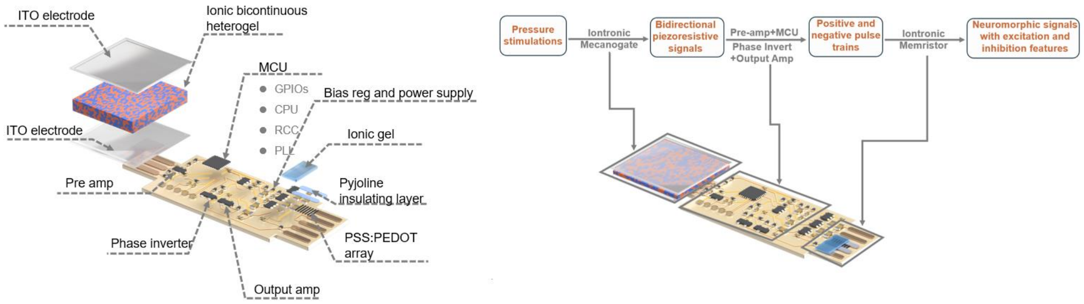

Supplementary Fig. 7 Mechanism and process of signal conversion in MIPM. A micro-integrated pre-amplifier, including GPIOs, CPU, RCC, and PLL-equipped microcontroller, inverter, output amplifier, and iontronic memristor were assembled on a flexible polyimide substrate. The pre-amplifier, connected to a mechanogate, receives and amplifies bidirectional piezoresistive signal inputs to the microcontroller, then generating the negative spike trains or positive spike trains. These positive and negative spike trains are further transmitted to downstream memristors to modulate the output of bipolarized neuromorphic signals.

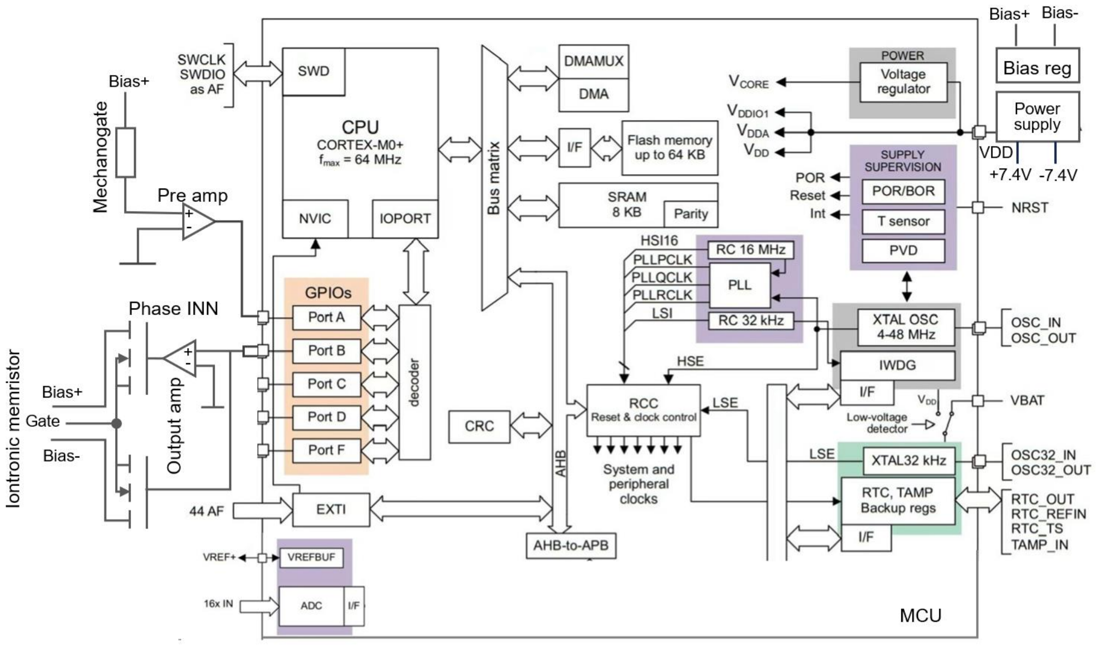

Supplementary Fig. 8 Design schematic for MIPM construction.

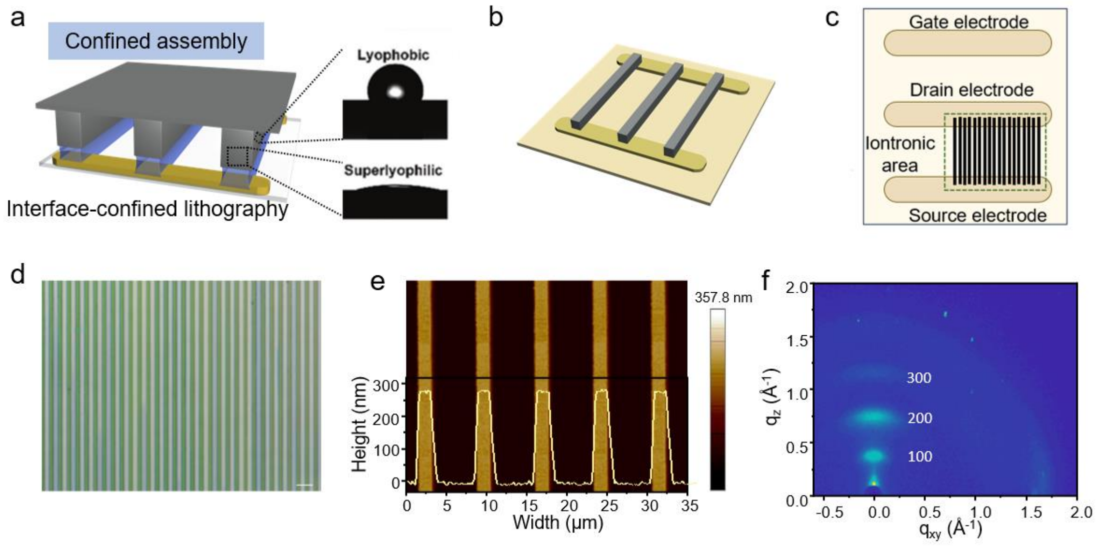

Supplementary Fig. 9 Preparation of high-quality PEDOT:PSS channels for memristors. **(a and b)** The schematic illustration of confined assembly of 1D arrays in the interface-confined assembly system. **(c)** Schematic diagram of the iontronic memristor structure with PEDOT:PSS array channels. (**d**) Microscopic image of the PEDOT:PSS channels. Scale bar, 10 μm. **(e)** AFM image and height diagram of PEDOT:PSS channels. **(f)** GIWAXS patterns of PEDOT:PSS channels, further suggesting the ordered crystallographic orientation of PEDOT:PSS.

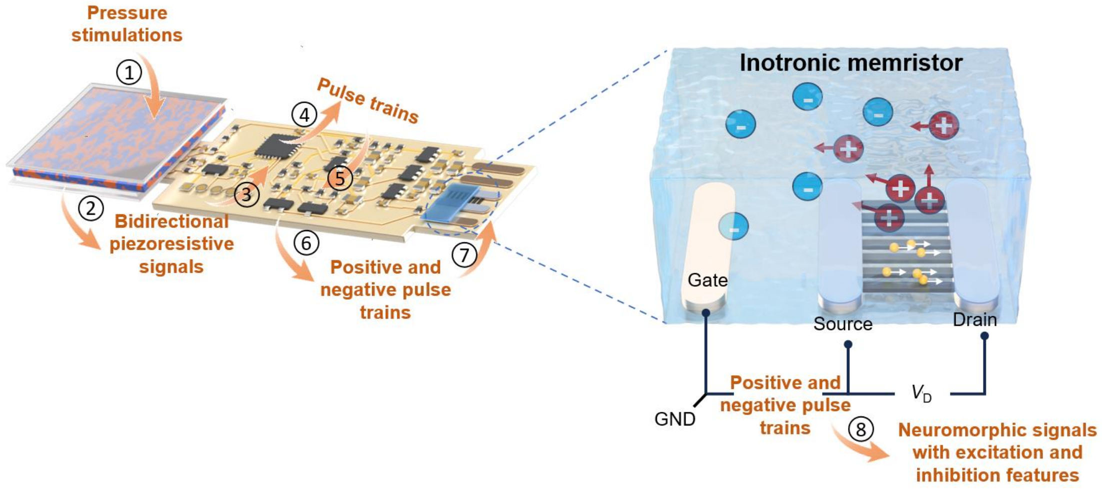

Supplementary Fig. 10 Mechanism and process of signal conversion in MIPM.

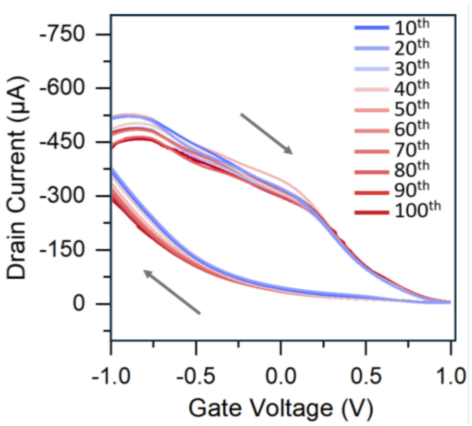

Supplementary Fig. 11 Current-voltage (*I*-*V*) curves of the memristor from the 10th to 100th cycle under ±1.0 V gate voltage sweep.

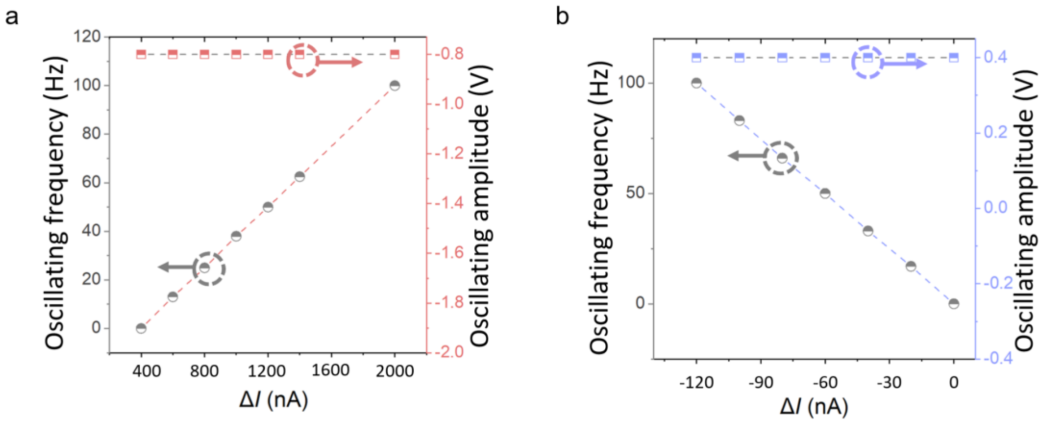

Supplementary Fig. 12 Current-oscillating frequency conversion curve of the MIPM at stiff and soft state, corresponding to positive (**a**) and negative (**b**) spike trains.

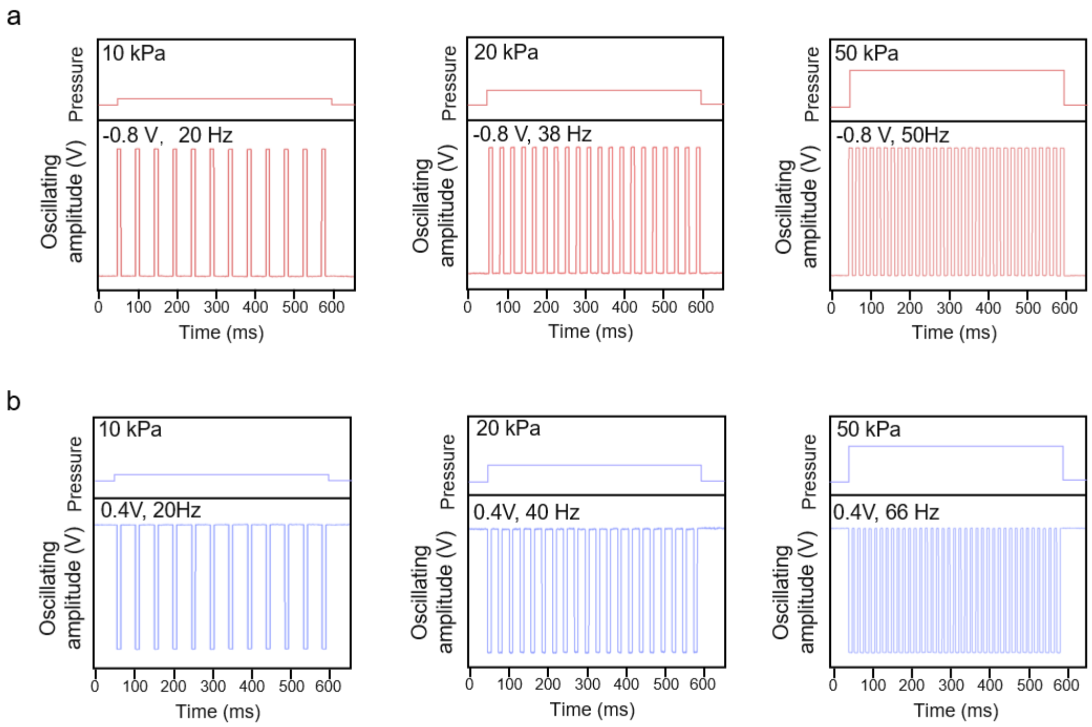

Supplementary Fig. 13 The pressure-induced **(a)** positive and **(b)** negative spike trains under different pressures (10, 20, and 50 kPa). The corresponding oscillating amplitudes and frequencies are labeled in each panel.

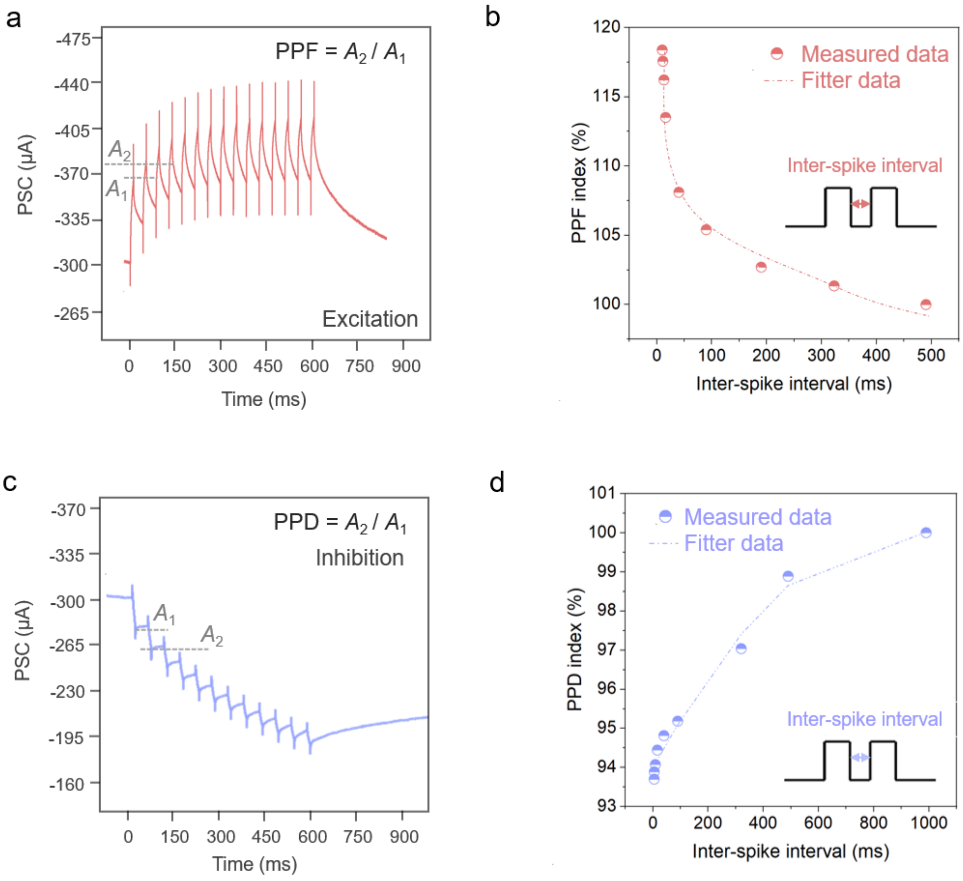

Supplementary Fig. 14 (a) Post-synaptic current (PSC) response showing paired-pulse facilitation (PPF) under 10 kPa in stiff state. **(b)** PPF indices as a function of inter-spike intervals. **(c)** PSC response showing paired-pulse depression (PPD) under 10 kPa in soft state. **(d)** PPD indices as a function of inter-spike intervals. The ratios A_2/A_1 are used to calculate the PPF and PPD indices. Insets show the corresponding stimulation patterns.

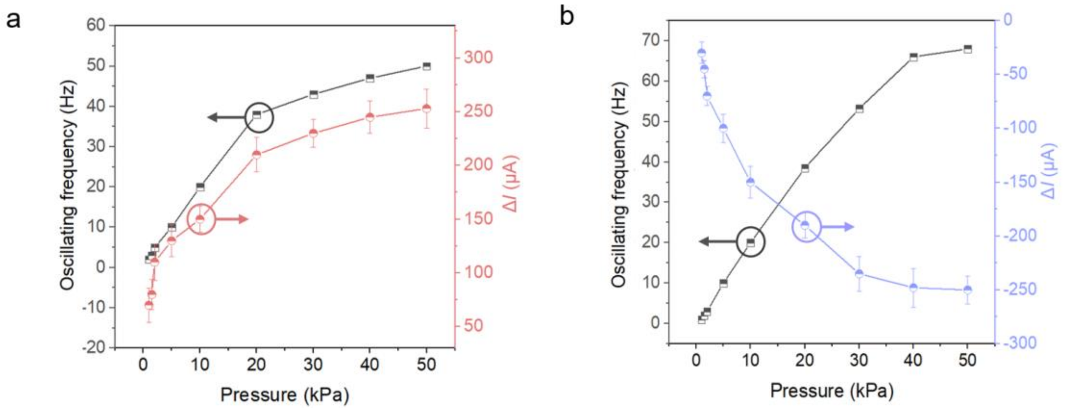

Supplementary Fig. 15 Pressure-dependent response characteristics of the MIPM in different states. **(a)** Oscillating frequency (black, left axis) and absolute postsynaptic current change ΔI (red, right axis) as a function of applied pressure in stiff state. **(b)** Oscillating frequency (black, left axis) and absolute postsynaptic current change ΔI (blue, right axis) as a function of applied pressure in soft state. Data points represent average values from ten measurements.

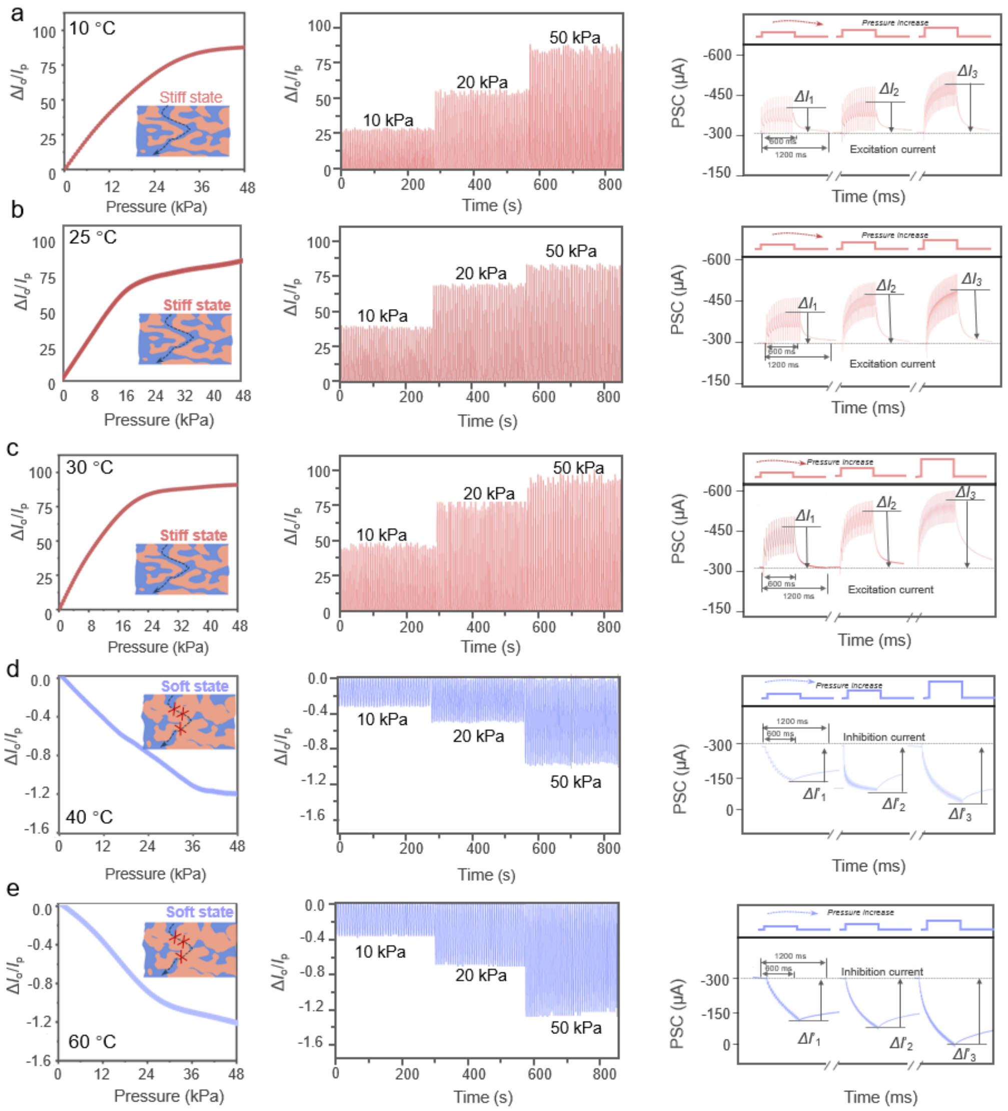

Supplementary Fig. 16 The piezoresistive properties ($\Delta I_O/\Delta I_P$) and postsynaptic current (PSC) signals of the MIPM system at **(a)** 10°C, **(b)** 25°C, **(c)** 30°C, **(d)** 40°C and **(e)** 60°C.

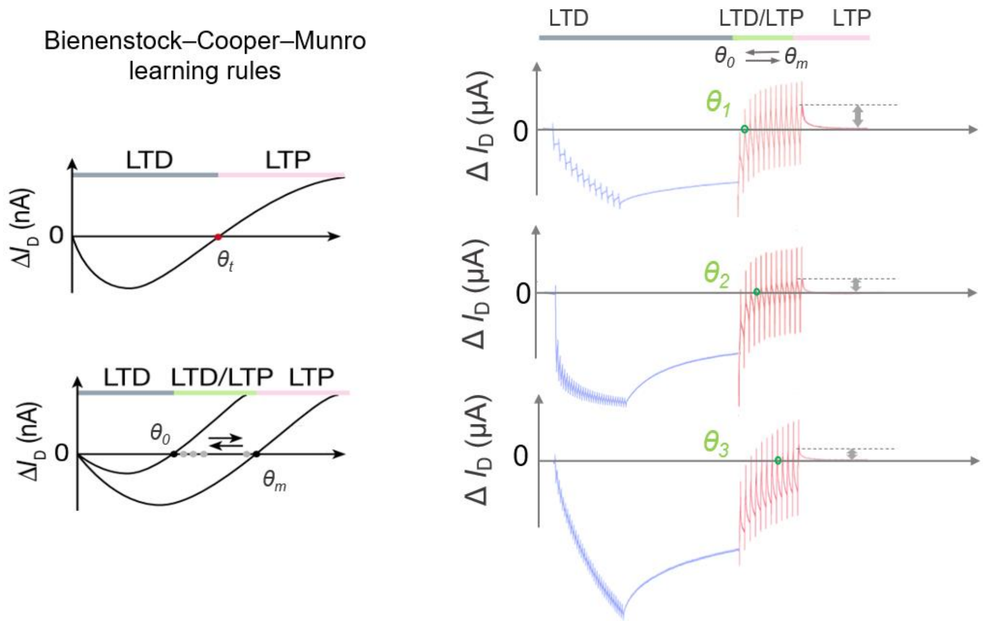

Supplementary Fig. 17 Bienenstock-Cooper-Munro learning rules.

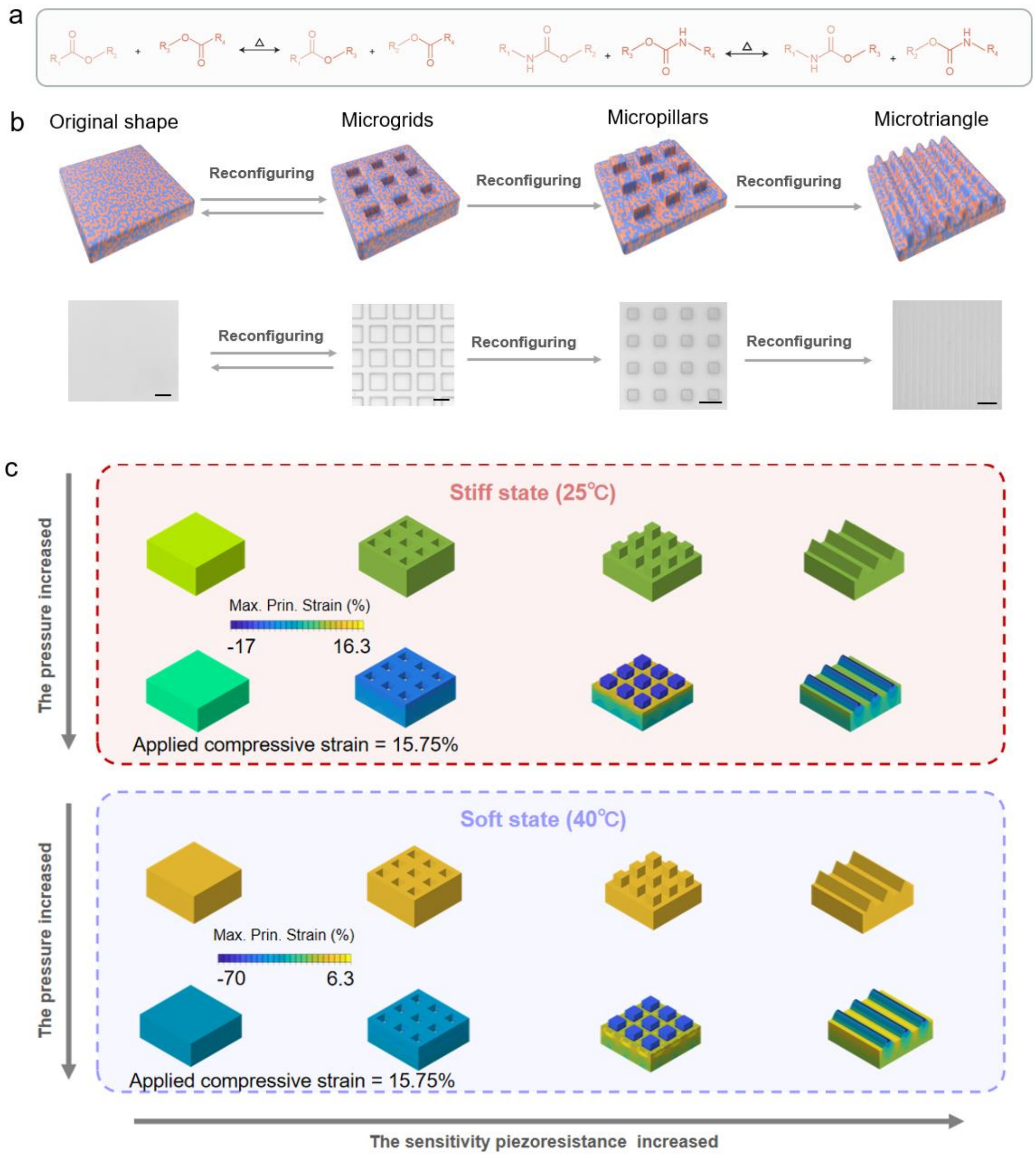

Supplementary Fig. 18 The shape reconfiguration of heterogels. **(a)** Schematic illustration of the dynamic covalent bonds in the vitrimer network. **(b)** Optical photographs of the heterogels with different microstructures. Scale bar, 100 μm. **(c)** Finite element simulations revealed varying strain responses in the microgrids, micropillars, and microtriangles, allowing the mechanogate to detect pressure with adjustable sensitivity at 25 and 40°C.

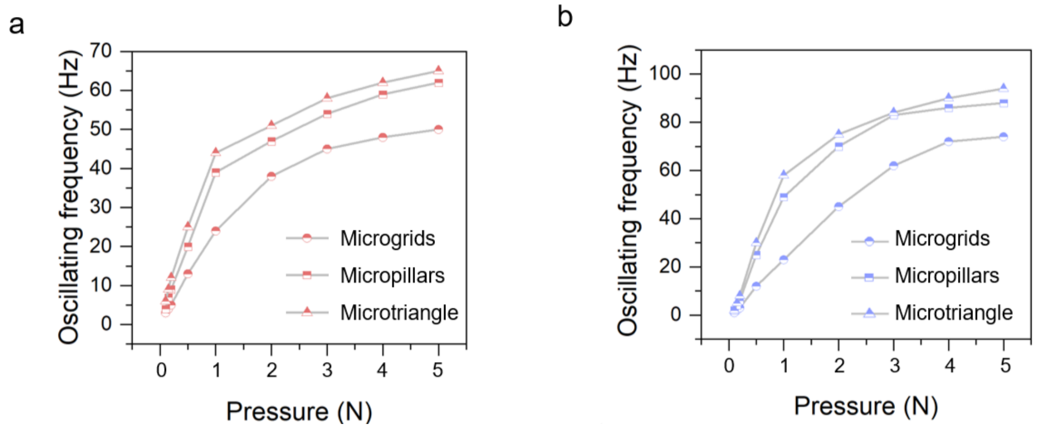

Supplementary Fig. 19 Pressure-dependent oscillating frequencies of MIPMs with microgrids, micropillars, and microtriangle microstructures in **(a)** stiff state (red curves) and **(b)** soft state (blue curves).

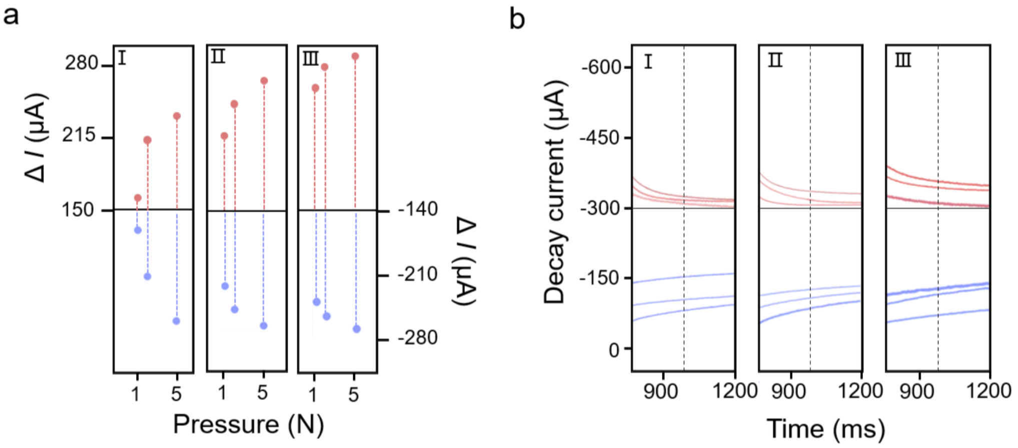

Supplementary Fig. 20 The absolute values of the change in postsynaptic current amplitudes **(a)** and decay current **(b)** of MIPM with microgrids (I), micropillars (II), and microtriangle (III) microstructures in stiff and soft state.

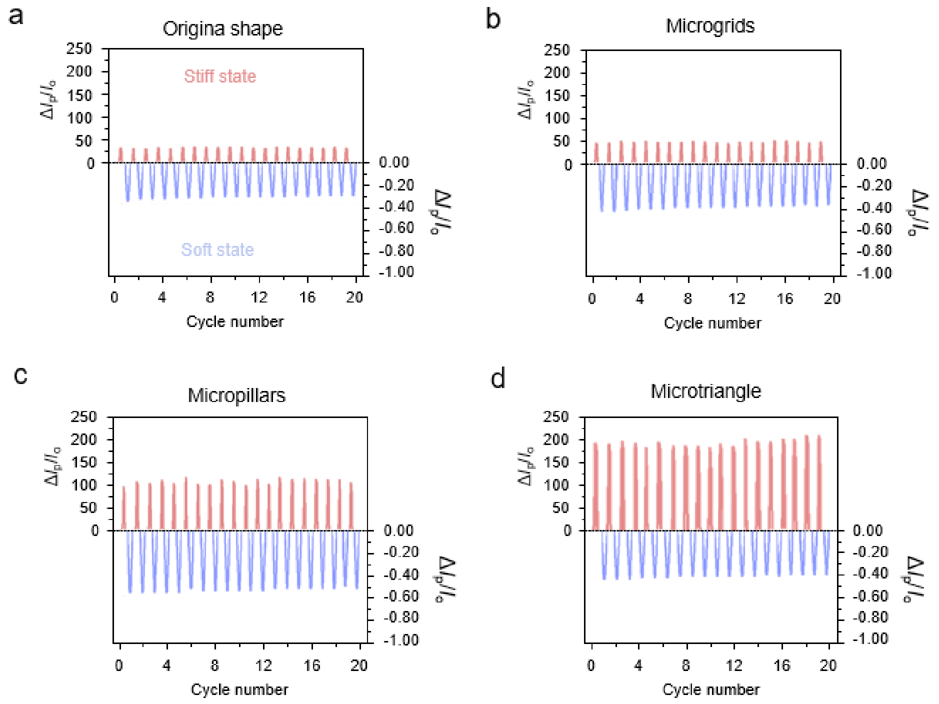

Supplementary Fig. 21 The stable bidirectional piezoresistive property of the heterogels with (a) original shape, (b) microgrids, (c) micropillars, and (d) microtriangle microstructures at switching cycles between stiff and soft states.

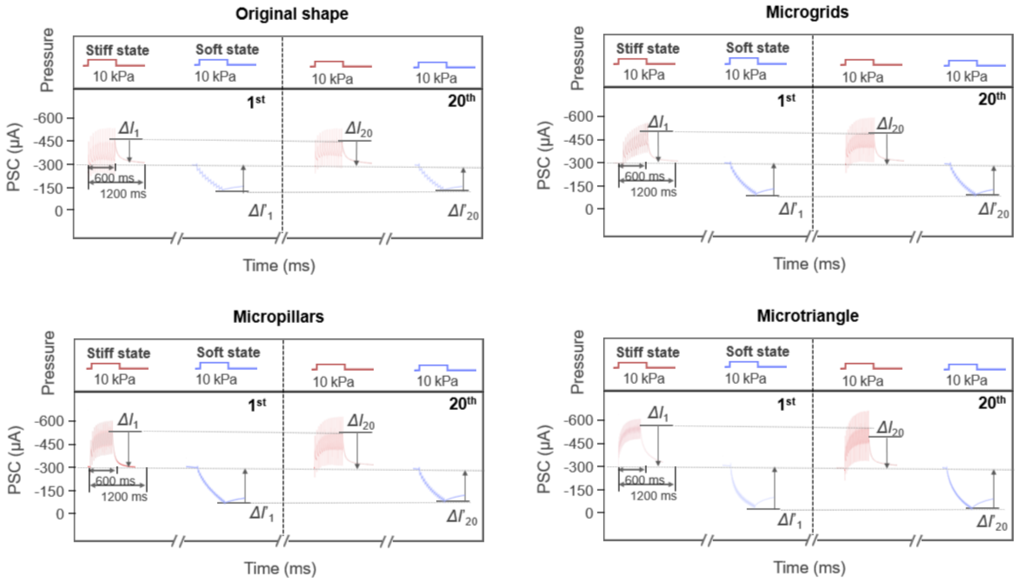

Supplementary Fig. 22 Comparison of postsynaptic current (PSC) responses for different microstructured MIPMs under same pressure stimulation. PSC traces showing the 1st and 20th cycles for **(a)** original shape, **(b)** microgrids, **(c)** micropillars, and **(d)** microtriangle microstructures. Each panel displays the stiff-gate (red) and soft-gate (blue) responses under 10 kPa pressure stimuli. ΔI_1 and ΔI_{20} represent the PSC amplitude changes in stiff state, while $\Delta I'_1$ and $\Delta I'_{20}$ represent the changes in soft state for the first and twentieth cycles, respectively.

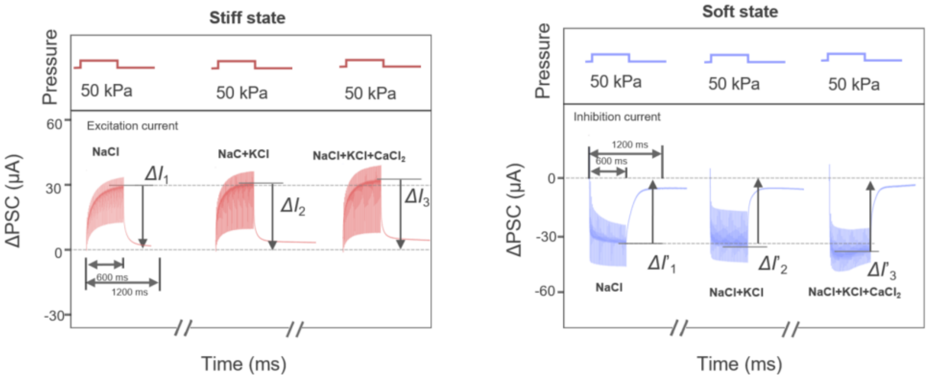

Supplementary Fig. 23 The post-synaptic current (PSC) of MIPMs corresponding 50 kPa loading pressure in different ion species state (maintaining equivalent total ionic concentrations 0.9%).

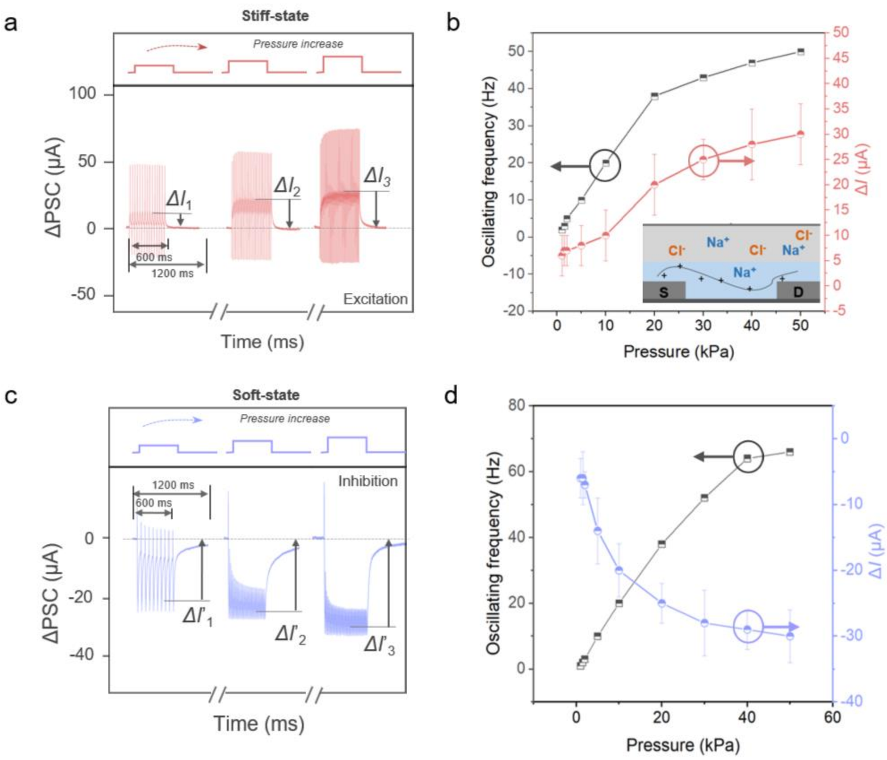

Supplementary Fig. 24 (a) Excitatory postsynaptic current (PSC) traces under sequential pressure stimuli in stiff state, showing increasing amplitude changes (ΔI_1, ΔI_2, ΔI_3). **(b)** Pressure-dependent oscillating frequency (black squares, left axis) and PSC amplitude changes (red circles, right axis) in stiff state with the single ion Na^+. **(c)** Inhibitory PSC traces under sequential pressure stimuli in soft state, showing amplitude changes ($\Delta I'_1$, $\Delta I'_2$, $\Delta I'_3$) with identical temporal parameters. **(d)** Pressure-dependent oscillating frequency (black squares, left axis) and PSC amplitude changes (blue circles, right axis) in soft state. Data points represent average values from ten measurements.

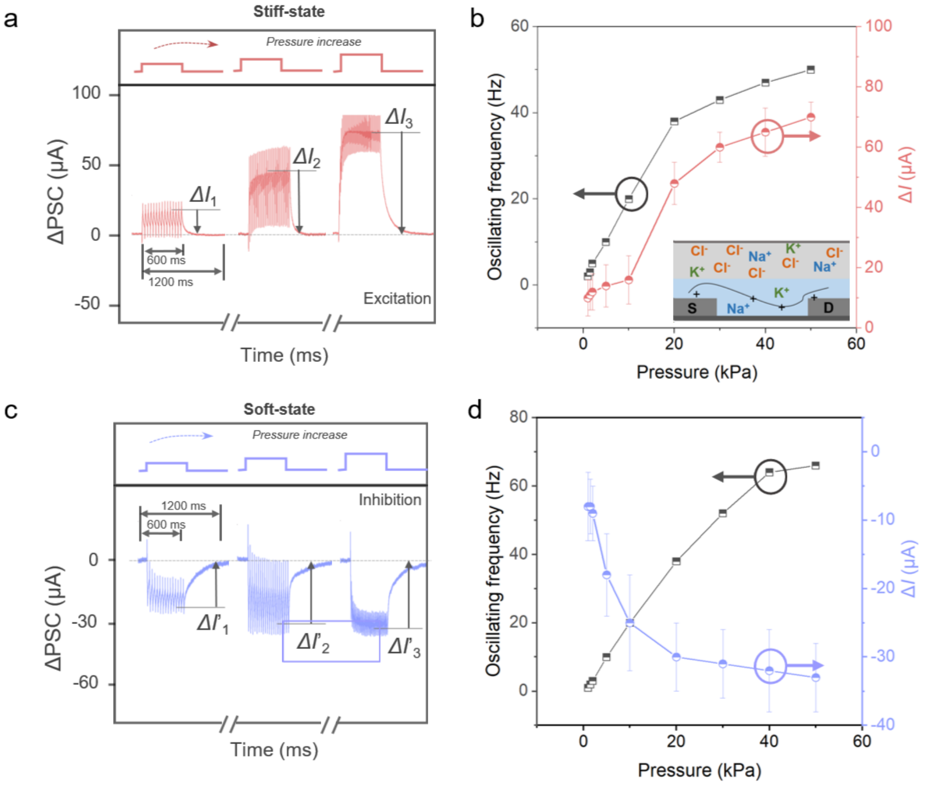

Supplementary Fig. 25 (a) Excitatory postsynaptic current (PSC) traces under sequential pressure stimuli in stiff state, showing increasing amplitude changes (ΔI_1, ΔI_2, ΔI_3). **(b)** Pressure-dependent oscillating frequency (black squares, left axis) and PSC amplitude changes (red circles, right axis) in stiff state with the multi-ion system including Na^+ and K^+. **(c)** Inhibitory PSC traces under sequential pressure stimuli in soft state, showing amplitude changes ($\Delta I'_1$, $\Delta I'_2$, $\Delta I'_3$) with identical temporal parameters. **(d)** Pressure-dependent oscillating frequency (black squares, left axis) and PSC amplitude changes (blue circles, right axis) in soft state. Data points represent average values from ten measurements.

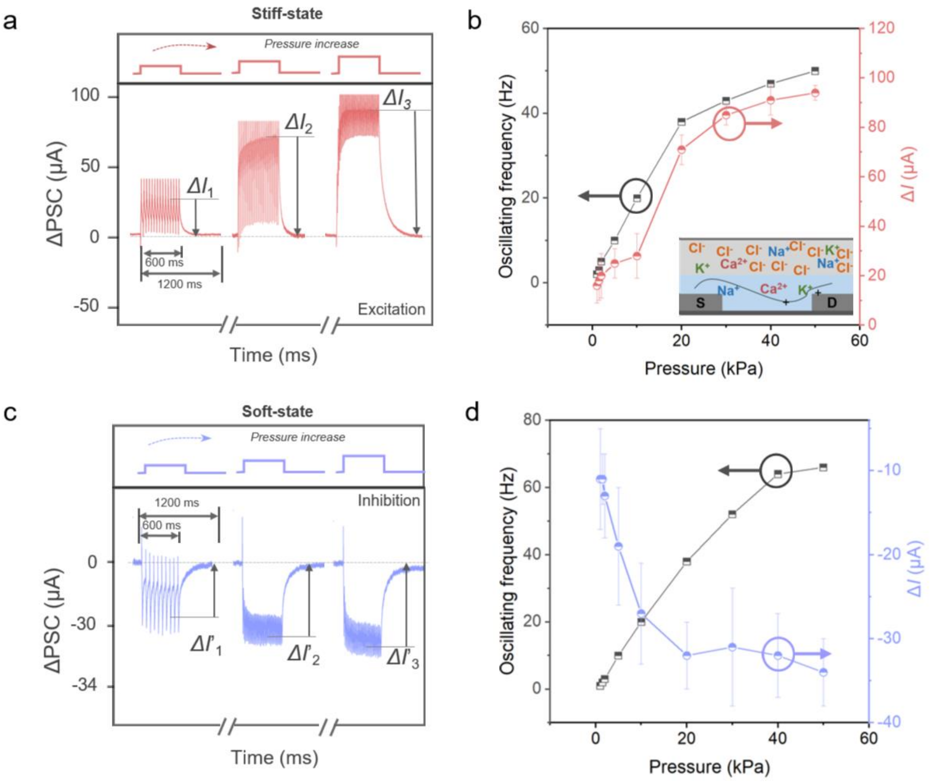

Supplementary Fig. 26 (a) Excitatory postsynaptic current (PSC) traces under sequential pressure stimuli in stiff state, showing increasing amplitude changes (ΔI_1, ΔI_2, ΔI_3). **(b)** Pressure-dependent oscillating frequency (black squares, left axis) and PSC amplitude changes (red circles, right axis) in stiff state with the multi-ion system including Na^+, K^+, and Ca^{2+}. **(c)** Inhibitory PSC traces under sequential pressure stimuli in soft state, showing amplitude changes ($\Delta I'_1$, $\Delta I'_2$, $\Delta I'_3$) with identical temporal parameters. **(d)** Pressure-dependent oscillating frequency (black squares, left axis) and PSC amplitude changes (blue circles, right axis) in soft state. Data points represent average values from ten measurements.

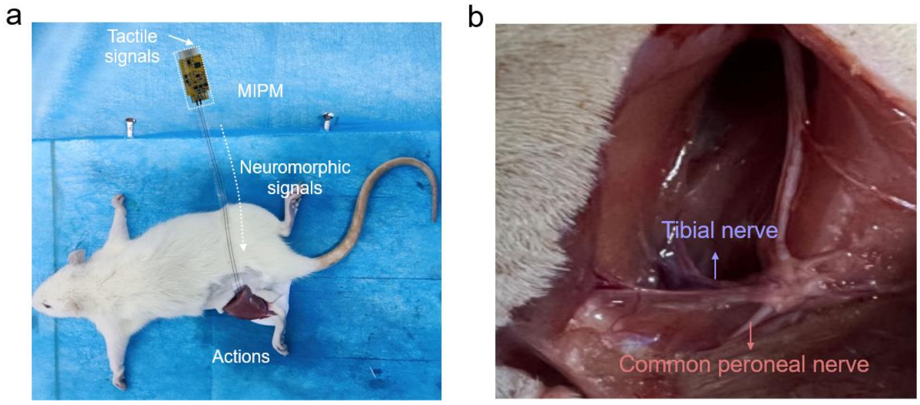

Supplementary Fig. 27 (**a**) Photograph of MIPM system for a biohybrid perception-actuation circuit. (**b**) The common peroneal and tibial nerves in the rat.

Supplementary Table 1. The statistics of piezoresistivity features in existing typical piezoresistive materials and the heterogels.

	Positive sensitivity (kPa^{-1})	Negative sensitivity (kPa^{-1})	Reference
Liquid metal	4.31	/	1
Ion hydrogel	2.83	/	2
	0.31	/	3
	1.33	/	4
Nanocompsites	5.28	/	5
	/	-0.0054	6
IL gel	2.45	/	7
Conductive polymers	4.50	/	8
Aerogel	1.02	/	9
	0.31	/	10
Liquid metal	/	-0.025	11
**Bicontinuous heterogels**	**4.34**	-0.034	**This work**

Supplementary References:

1. Yun, G. et al. Liquid metal hybrid composites with high-sensitivity and large dynamic range enabled by micro- and macrostructure engineering. *ACS Appl. Polym. Mater.* **3**, 5302-5315 (2021).

2. Han, X. et al. Green and stable piezoresistive pressure sensor based on lignin-silver hybrid nanoparticles/polyvinyl alcohol hydrogel. *Int. J. Biol. Macromol.* **176**, 78-86 (2021).

3. Shen, J. et al. A bioinspired porous-designed hydrogel polyurethane sponge piezoresistive sensor for human−machine interfacing. *Nanoscale* **13**, 19155-19163 (2021).

4. Yang, H. et al. Coupling thermogalvanic and piezoresistive effects in a robust hydrogel for Deep-Learning-Assisted Self-Powered sign language and object recognition. *Chem. Eng. J.* **488**, 150816 (2024).

5. Park, S. et al. Piezo-impedance response of carbon nanotube/polydimethylsiloxane nanocomposites. *APL Mater.* **7**, 041118 (2019).

6. Liu, H. et al. Ambilateral convergent directional freeze casting meta-structured foams with a negative Poisson's ratio for high-performance piezoresistive sensors. *Chem. Eng. J.* **454**, 140436 (2023).

7. Mogli, G. et al. Ultrasensitive piezoresistive and piezocapacitive cellulose-based ionic hydrogels for wearable multifunctional sensing. *ACS Appl. Electron. Mater.* **5**, 205-215 (2023).

8. Teixeira, J. et al. Lanceros-méndez, Piezoresistive response of extruded polyaniline/(styrene-butadiene-styrene) polymer blends for force and deformation sensors. *Mater. Des*. **141**, 1-8 (2018).

9. Wang, Y. et al. Lightweight and elastic silver nanowire/PEDOT:PSS/polyimide aerogels for piezoresistive sensors. *ACS Appl. Polym. Mater.* **4**, 3205-3216 (2022).

10. Kang, W. et al. Photocrosslinked methacrylated carboxymethyl chitin hydrogels with tunable degradation and mechanical behavior. *Carbon* **113**, 395-403 (2017).

11. Yun, G. et al. Liquid metal composites with anisotropic and unconventional piezoconductivity. *Matter* **3**, 824-841 (2020).
