# Supplementary material for: Mechano-gated iontronic piezomemristor for temporal-tactile neuromorphic plasticity
Source: Nat Commun. 2025 Jan 26;16:1060. doi: 10.1038/s41467-025-56393-w (PMC11770186; doi:10.1038/s41467-025-56393-w)
Supplement: Supplementary file 2 — Description of Additional Supplementary Files [file 41467_2025_56393_MOESM2_ESM.pdf]

Description of Additional Supplementary Files

File Name: Supplementary Movie 1
Description: A Mechano-gated Iontronic Piezomemristor for a biohybrid perception-actuation circuit.
